# The Mediating Role of Resilience and Extraversion on Psychological Distress and Loneliness Among the General Population of Tyrol, Austria Between the First and the Second Wave of the COVID-19 Pandemic

**DOI:** 10.3389/fpsyt.2021.766261

**Published:** 2021-10-27

**Authors:** Anna Chernova, Beatrice Frajo-Apor, Silvia Pardeller, Franziska Tutzer, Barbara Plattner, Christian Haring, Bernhard Holzner, Georg Kemmler, Josef Marksteiner, Carl Miller, Martin Schmidt, Barbara Sperner-Unterweger, Alex Hofer

**Affiliations:** ^1^Division of Psychiatry I, Department of Psychiatry, Psychotherapy and Psychosomatics, Medical University Innsbruck, Innsbruck, Austria; ^2^Department of Psychiatry, Sanitary Agency of South Tyrol, General Hospital of Bolzano, Bolzano, Italy; ^3^Department of Psychiatry and Psychotherapy B, State Hospital Hall in Tyrol, Hall in Tyrol, Austria; ^4^Department of Psychiatry and Psychotherapy A, State Hospital Hall in Tyrol, Hall in Tyrol, Austria; ^5^Department of Psychiatry, County Hospital Kufstein, Kufstein, Austria; ^6^Department of Psychiatry, County Hospital Lienz, Lienz, Austria; ^7^Division of Psychiatry II, Department of Psychiatry, Psychotherapy and Psychosomatics, Medical University Innsbruck, Innsbruck, Austria

**Keywords:** COVID-19, psychological distress, loneliness, resilience, extraversion, mental health

## Abstract

**Background:** During the first 3 weeks of the COVID-19 pandemic, the federal state of Tyrol, Austria had one of the strictest curfews in Austria and worldwide. The aim of the current study was to investigate the assumingly protective role of resilience and extraversion and its impact on mental health following such an uncertain and unpredictable situation.

**Methods:** Between the first and the second wave of the pandemic, adult residents of Tyrol were invited to participate in an online survey. Next to the assessment of sociodemographic and COVID-19-related variables the Brief-Symptom-Checklist, the Three-Item Loneliness Scale, the Resilience Scaled, and the Big Five Inventory were used to assess psychological distress, loneliness, resilience, and extraversion. Mediation analysis was used to investigate the role of resilience and extraversion in the context of age-, sex-, and partnership- related differences in psychological distress and loneliness.

**Results:** One hundred and forty-five participants took part in the survey (68.2% female). Overall, psychological distress and severe loneliness were more often detected in women and singles. They also were less resilient, while men and singles presented with a lower degree of extraversion. Study participants under the age of 30 experienced severe loneliness more frequently than older people, whereas psychological distress, resilience, and extraversion were comparable between age groups. Resilience significantly mediated the relationship between both study participants' sex and partnership situation on one hand and psychological distress and severe loneliness on the other. In addition, extraversion significantly mediated the relationship between participants' partnership situation and psychological distress.

**Discussion:** Our findings suggest that women, singles, and young people may be particularly affected by the measures and sequelae of the COVID-19 pandemic. Interventions promoting resilience and extraversion among these groups are urgently needed to foster mental health. Ideally, they can be utilized at home in case of renewed mobility restrictions or quarantine in the future.

## Introduction

The COVID-19 pandemic reached Tyrol, Austria as one of the first regions in Europe in March 2020 (1). As declared by the Islandic authorities on March 5th, the ski resort of Ischgl ranked as a risk region at the beginning of the pandemic (2). Initially, the Austrian government reacted with quarantine measures for the Ischgl area, and subsequently for entire Tyrol, followed by a first nation-wide lockdown on March 16th (1). The special aspect of the measures in Tyrol was that no one was allowed to leave the house without a compelling reason, but only for buying groceries, to get to the workplace, or to assist care-dependent others (3). Even going for a walk at a distance of one meter was not allowed in Tyrol (3, 4), but in the rest of Austria (5). This quarantine in Tyrol ended on April 7th, and the first lockdown in Austria on May 1st, 2020 (6). The governmental relaxations in May and June 2020 initially raised the hope for the return to normality and the defeat of the virus. However, it is known that quarantine, as an unexpected intervention in everyday life, can have negative psychological impacts (7). All over the world, the prevalence of psychological distress (8–10) and loneliness (11, 12) has increased in the context of the pandemic. Moreover, boredom is a major issue during lockdown that particularly affects women, singles, unemployed, and low-income people (13). In this context, it is important to foster protective factors that can contribute to maintaining psychological stability. Next to coping mechanisms and social support, the overall construct of resilience is deemed to be relevant in this context.

The concept of resilience describes a dynamic system to cope with adverse life events and stress (14) as well as the ability to quickly balance, recover, and return to a healthy initial state (15). It is still a young concept (15) and to date, there is no uniform definition of resilience (16). Generally, resilience is known to protect from psychological distress (17) and loneliness (18). Moreover, higher levels of resilience are associated with better psychological well-being (17, 19), lower levels of anxiety (17) and depression (17, 20–22), a decreased likelihood of posttraumatic stress disorder (23), and less stress (24). These are important factors in managing the COVID-19 pandemic. Thus, we hypothesized that people with higher resilience are less stressed by the pandemic, the countermeasures, and their sequelae. Next to resilience, certain personality traits could also have an influence on coping strategies (25). Extraversion seems to be particularly important in this context, as extroverts are particularly in need of closeness and contact to other people.

Extraversion is a personal trait describing active people who are sociable, talkative, and assertive (26). These people tend to be outgoing, prone to establish social contacts, seeking for closeness (27), and thus, they prefer large groups and gatherings (25). They are likely to have high self-esteem (28), and to experience peculiar and complex events with lower stress levels and more positive feelings (29). Examining extraversion in relation to psychological distress and loneliness is important, because there were no curfews to this large extent in times of peace prior to COVID-19. Since previous research has shown that people with higher extraversion are inert to stress (30), we hypothesized that people with higher levels of extraversion were less psychologically distressed after the first lockdown.

Most previous studies on psychological distress and loneliness during the COVID-19 pandemic focussed predominantly on the time period during lockdown. However, our online survey was done in the period between the first and the second lockdown in Austria, when governmental restrictions were softened. The aim of the current study was to investigate the assumingly protective role of resilience and extraversion and its impact on mental health following such an uncertain and unpredictable situation.

## Methods

Focussing on the general population of Tyrol, Austria (approximately 760,000 inhabitants), we used a web-based, cross-sectional survey to evaluate the associations between resilience and extraversion and their impact on psychological distress and loneliness amidst the COVID-19 pandemic. The survey was conducted between June 26th and September 13th, 2020.

Data collection was performed in an anonymized manner using the web-based software program Computer-based Health Evaluation System (CHES) (31). Next to the collection of sociodemographic and COVID-19-related data psychological distress, loneliness, resilience, and extraversion were investigated by using online questionnaires (see below).

Members from the general population of Tyrol aged above 18 were invited to participate in the study through advertising in both print and social media. Online consent was obtained at the beginning of the survey and participants were asked to provide an email address in order to be reminded for follow-up surveys. Provision of email addresses was not a prerequisite to participate in the baseline survey. At the end of the survey, participants received a downloadable information sheet on professional support numbers and addresses. Ethical approval was obtained from the ethics committee of the Medical University Innsbruck.

### Sociodemographic and COVID-19-Related Data

Data on a variety of demographic aspects were collected including age, gender, education, employment status, professional field, household income, marital and parental status, living situation as well as personal and family history of psychiatric disorders. In addition, some COVID-19-related questions were included, e.g., whether participants had been tested for COVID-19, whether some of their relatives had been tested positive, and whether the measures to prevent the spread of the virus were considered as useful.

### Psychological Distress

The Brief-Symptom-Checklist (BSCL) (32) was used to evaluate participants' subjectively perceived impairment through 53 physical and psychological symptoms. Items were rated on a 5-point Likert scale (0 = not at all, 4 = extremely). The BSCL quantifies nine symptom dimensions: somatization, obsession-compulsion, interpersonal sensitivity, depression, anxiety, hostility, phobic anxiety, paranoid ideation, and psychoticism. A Global Severity Index (GSI) is calculated using the sums of the nine symptom dimensions plus four additional items not included in any of the dimension scores divided by the total number of answered items. Based on community norms, a GSI T-score ≥63 was used as a cut-off score to indicate significant distress. The BSCL has shown good to satisfactory internal consistency for all subscales (Cronbach's α ranging from 0.70 to 0.89) and excellent external consistency for the GSI score (α = 0.96) (33).

### Loneliness

Loneliness was assessed by using the Three-Item Loneliness Scale (TILS) (34), which is known to demonstrate acceptable internal consistency (Cronbach's α = 0.72). The TILS represents an abbreviated form of the Revised University of California Los Angeles (R-UCLA) Loneliness Scale (35). Participants are asked “How often do you feel that you lack companionship?”, “How often do you feel left out?”, and “How often do you feel isolated from others?”. Possible answers are “often” (scored 1), “some of the time” (scored 2), and “hardly ever or never” (scored 3). The summary score ranges from 3 to 9 points, higher scores suggest greater loneliness. A TILS-score ≥7 was considered to indicate severe loneliness.

### Resilience

Resilience was evaluated using the Resilience Scale (RS-13) (36), a revised short form of the RS-25 (37) with a good internal consistency of Cronbach's α = 0.90 (36). It consists of 13 items scored on a 7-point scale, ranging from 1 = strongly disagree to 7 = strongly agree. Possible scores range from 13 to 91, higher scores indicate higher resilience. A score up to 66 reflects low resilience, scores between 67 and 72 indicate moderate resilience, and scores of 73 and higher indicate high resilience.

### Extraversion

The Big Five Inventory (BFI) (38) is a 44-item self-administered questionnaire, which measures the five personality traits extraversion, openness, conscientiousness, agreeableness, and neuroticism on a 5-point Likert scale ranging from 1 = strongly disagree to 5 = strongly agree. The German version used in this study has been validated by Lang et al. (39). We exclusively applied the extraversion subscale (8 questions), which has a good internal consistency (Cronbach's α = 0.90) (39). Possible scores range from 8 to 40, higher scores indicate more pronounced extraversion.

### Statistical Methods

All statistical analyses were performed using SPSS, version 26. Sociodemographic and health-related sample characteristics were described by simple summary statistics, means, standard deviations, relative frequencies, etc. The main focus of the analysis was placed on psychological distress and loneliness as the primary outcome variables and on resilience and extraversion as potentially protective factors. Psychological distress and loneliness were dichotomized for the analysis (GSI T-score ≥63 vs. <63, TILS total score ≥7 vs. <7, respectively), whereas resilience (RS-13 total) and extraversion (BFI subscale Extraversion) were used as continuous scales. Group differences in psychological distress and severe loneliness with regard to age, sex, and partnership were analyzed by means of Chi-square tests, using odds ratios to quantify effect sizes. Due to the skewed distribution of resilience and extraversion, group differences in these variables were analyzed by non-parametric methods (Mann–Whitney *U*-test, Kruskal–Wallis test).

To investigate the relationship between the above variables in more detail we performed several mediation analyses both for the dependent variables psychological distress and severe loneliness (variable Y). The variables sex (male, female), age group (three groups) and partnership (yes/no) served as independent variables (variable X). Resilience and extraversion were regarded as potential mediators, testing for the significance of their effect (variable M). Group variables that were not used in a particular analysis were included as covariates to control for their effect. In each of the mediation analyses, the total effect of X on Y was split up into a direct effect of X on Y and a mediation effect, where the latter represented the part that is accounted for by the mediators via the path X → M → Y. For model fitting and parameter estimation, we applied the PROCESS macro developed by Hayes, using the mediation model no. 4 (40). Significance was confirmed by the Sobel *Z*-test and bootstrapping with 5,000 bootstrap samples. All continuous variables were z-standardized prior to the mediation analysis.

## Results

A sample of 1,045 people from the general population of Tyrol participated in the study. Mean age was 41.4 ± 14.0 years, 68.2% were female 68.6% had a full-time or part-time employment, and the majority (74.4%) were in a permanent partnership. At the time of the survey, 10.4% of respondents were in psychological/psychotherapeutic treatment, and 6.9% in psychiatric treatment. Sociodemographic characteristics of the study sample and health-related variables are presented in Table 1.

**Table 1:** Sociodemographic and health-related variables (*N* = 1,045).

**Variable**	**Mean ± SD or *N* (%)**
Sex	
Male	331 (31.7%)
Female	713 (68.2%)
Others	1 (0.1%)
Age (Years)	41.4 ± 14.0 (18–96)
Education (Years)	15.5 ± 3.8 (8–30)
Partnership	
Single	267 (25.6%)
Permanent partnership	777 (74.4%)
Children in same household	
None	696 (66.8%)
1	142 (13.2%)
2	158 (15.2%)
≥3	46 (4.4%)
Work situation	
Full time or part-time employment	716 (68.6%)
Self-employed	45 (4.3%)
Education/training	74 (7.1%)
Home office	13 (1.2%)
Short-time work	26 (2.5%)
Unemployed	12 (1.1%)
Retired	97 (9.3%)
Homemaker	19 (1.8%)
Others	42 (4.0%)
Household income	
<25,000 €/year	392 (37.5%)
25,000–49,999 €/year	387 (37.0%)
≥50,000 €/year	235 (22.5%)
Not specified	31 (3.0%)
Place of residence	
Urban (Innsbruck > 100,000 inhabitants)	346 (33.0%)
Village or small town	640 (61.1%)
Places with high exposition to COVID-19	50 (4.8%)
Not specified	9 (0.9%)
Severe physical health problems	90/1,043 (8.6%)
Mental health problems, lifetime	181/1,043 (17.4%)
Current psychiatric treatment	72/1,043 (6.9%)
Current psychological/psychotherapeutic treatment	108/1,043 (10.4%)
Psychological distress [GSI T-Score (BSCL) ≥63]	145/998 (14.4%)
Severe loneliness (TILS score ≥7)	223/1,004 (22.2%)
Resilience (RS-13 total score)	71.7 ± 12.3
Extraversion (BFI total score)	27.8 ± 5.8
SARS-CoV-2 test	
No test performed	742 (71.0%)
Negative test result	274 (26.2%)
Positive test result	23 (2.2%)
Result unknown/not specified	6 (0.6%)
Severity of COVID-19 Symptoms (*n* = 23)	
No symptoms	5 (21.7%)
Mild symptoms	10 (43.5%)
Symptoms with fever, treatment at home	7 (30.4%)
Severe symptoms, treatment in hospital	1 (4.3%)

*BSCL, Brief-Symptom-Checklist; TILS, Three Item Loneliness Scale; RS-13; Resilience Scale; BFI, Big Five Inventory*.

### Differences in Psychological Distress, Loneliness, Resilience, and Extraversion Between Subgroups

Differences in psychological distress, loneliness, resilience, and extraversion between subgroups are displayed in Table 2. With regard to sex, significantly more women than men reported psychological distress (16.2 vs. 10.4%). Women were also twice as likely to report severe loneliness (TILS score ≥7) (26 vs. 13.5%). In the RS-13, men indicated a significantly higher degree of resilience with a mean of 73.4 ± 11.5 points compared to a mean of 70.9 ± 12.7 points in women. In turn, a mean of 28.1 ± 5.9 points in the BFI indicates that women were significantly more extraverted than men (27.3 ± 5.6 points).

Table 2Differences in psychological distress, loneliness, resilience, and extraversion between subgroups (sex, partnership situation, and age group).

**Table:** 

**Grouping variable**		**Group 1**	**Group 2 (reference)**	**Comparison**		
**Sex**		**Female**	**Male**	**Effect size**	**Statistics^a^**	***p*-value**
Psychological distress	% (*N*)	16.2% (113/689) ↑	10.4% (32/308)	OR = 1.69	χ^2^ = 6.19	0.013
Severe loneliness	% (*N*)	26.0% (180/692) ↑	13.5% (42/311)	OR = 2.52	χ^2^ =19.47	<0.001
Resilience	Mean ± SD	70.9 ± 12.7 ↓	73.4 ± 11.5	*d* = −0.20	*Z* = −3.02	0.003
Extraversion	Mean ± SD	28.1± 5.9 ↑	27.3 ± 5.6	*d* = 0.14	*Z* = 2.12	0.034
**Partnership situation**		**No partnership**	**Partnership**	**Effect size**	**Statistics**	***p*** **-value**
Psychological distress	% (*N*)	22.1% (57/258) ↑	11.9% (88/740)	OR = 2.10	χ^2^ = 16.03	<0.001
Severe loneliness	% (*N*)	33.3% (86/258) ↑	18.4% (137/746)	OR = 2.22	χ^2^ = 24.86	<0.001
Resilience	Mean ± SD	68.6 ± 13.8 ↓	72.7 ± 11.6	*d* = −0.34	*Z* = −4.07	<0.001
Extraversion	Mean ± SD	27.1 ± 6.2 ↓	28.1 ± 5.6	*d* = −0.17	*Z* = −2.00	0.048

**Table:** 

**Age group**		**Group 1** **(18–29 years)**	**Group 2** **[30–59 years (ref.)]**	**Group 3** **(60–96 years)**	**Effect size**	**Statistic**	***p*** **-value**
Psychological distress	% (*N*)	15.6% (38/244)	14.3% (92/645)	13.1% (14/107)	OR_1_ = 1.11^b^ OR_3_ = 0.90^c^	χ^2^ = 0.43	0.919^n.s.^
Severe loneliness	% (*N*)	28.2% (69/245)↑	20.9% (136/650)	15.9% (17/107)	OR_1_ = 1.48^b^ OR_3_ = 0.71^c^	χ^2^ = 8.14	0.017
Resilience	Mean ± SD	72.1 ± 11.2	71.8 ± 12.5	70.3 ± 13.7	η^2^ = 0.002	χ^2^ = 0.532	0.874^n.s.^
Extraversion	Mean ± SD	28.4 ± 5.8	27.6 ± 5.8	27.5 ± 5.8	η^2^ = 0.003	χ^2^ = 3.381	0.184^n.s.^

*SD, Standard deviation; OR, odds ratio; d, Cohen's effect size d; η^2^, partial eta squared; n.s., not significant (p > 0.05)*.

*↑significantly higher than in group 2 ↓ significantly lower than in group 2*.

a *Psychological distress (yes/no) and severe loneliness (yes/no) were analyzed by means of logistic regression, resilience and extraversion were analyzed by Mann–Whitney U-Test (two-group comparisons) and Kruskal–Wallis test (three-group comparisons)*.

b *OR_1_, Odds ratio age group (1) vs. reference age group (2)*.

c *OR_3_, Odds ratio age group (3) vs. reference age group (2)*.

When using partnership situation as a grouping variable, a significantly higher proportion of singles reported psychological distress (22.1%) and severe loneliness (33.3%) compared to study participants living in a permanent partnership (11.9 and 18.4%, respectively). Similarly, singles indicated a significantly lower degree of resilience compared to those living in a permanent partnership (mean of 68.6 ± 13.8 vs. 72.7 ± 11.6 points in the RS-13) and were significantly less extraverted (mean of 27.1 ± 6.2 vs. 28.1 ± 5.6 points in the BFI).

Age was divided into three groups. Psychological distress, resilience, and extraversion were comparable between age groups, whereas, severe loneliness was significantly more frequently observed in the group aged 18–29 years (28.2%) compared to the groups aged 30–59 years (20.9%) and 60–96 years (15.9%).

### Association of Resilience and Extraversion With Psychological Distress and Severe Loneliness

We found a positive interrelation between resilience and extraversion. These two constructs were negatively associated with both psychological distress and severe loneliness (Table 3).

**Table 3:** Association of resilience and extraversion with psychological distress and severe loneliness (Spearman rank correlation coefficients).

		**Extraversion** **(BFI total)**	**Psychological distress** **[GSI T-score** **(BSCL) ≥ 63]**	**Severe loneliness** **(TILS ≥ 7)**
Resilience RS-13 total	Spearman rho	0.407^**^	−0.310^**^	−0.214^**^
	*p*-value	<0.001	<0.001	<0.001
	*N*	1,005	997	1,003
Extraversion BFI total	Spearman rho	–	−0.201^**^	−0.068^*^
	*p*-value	–	<0.001	0.032
	*N*	–	996	1,002

*BFI, Big Five Inventory; BSCL, Brief-Symptom-Checklist; TILS, Three Item Loneliness Scale; RS-13, Resilience Scale*.

* *p < 0.05*;

** *p < 0.01*.

### Results of Mediation Analyses

**Figure 1:**
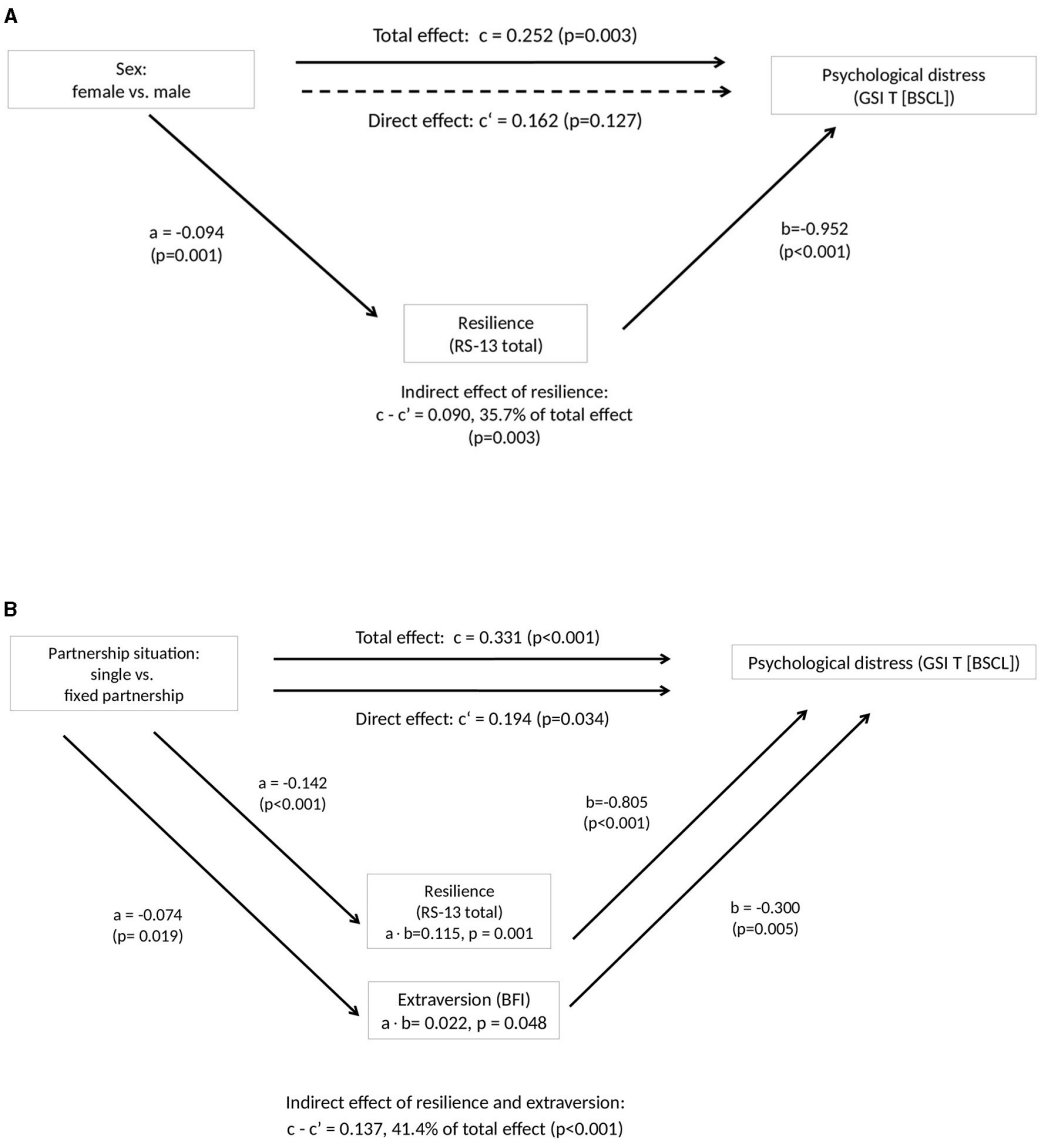
Findings of mediation analyses 1, dependent variable psychological distress. **(A)** Effect of resilience as a mediator on the relationship between sex and psychological distress. Numbers shown are standardized regression coefficients. Solid lines indicate statistically significant effects, dashed lines indicate non-significant effects. Extraversion (BFI total score) did not show a significant mediation effect. **(B)** Indirect effect of resilience and extraversion on the relationship between partnership situation and psychological distress. Numbers shown are standardized regression coefficients. Solid lines indicate statistically significant effects. BSCL, Brief-Symptom-Checklist; RS-13, Resilience Scale; BFI, Big Five Inventory.

**Figure 2:**
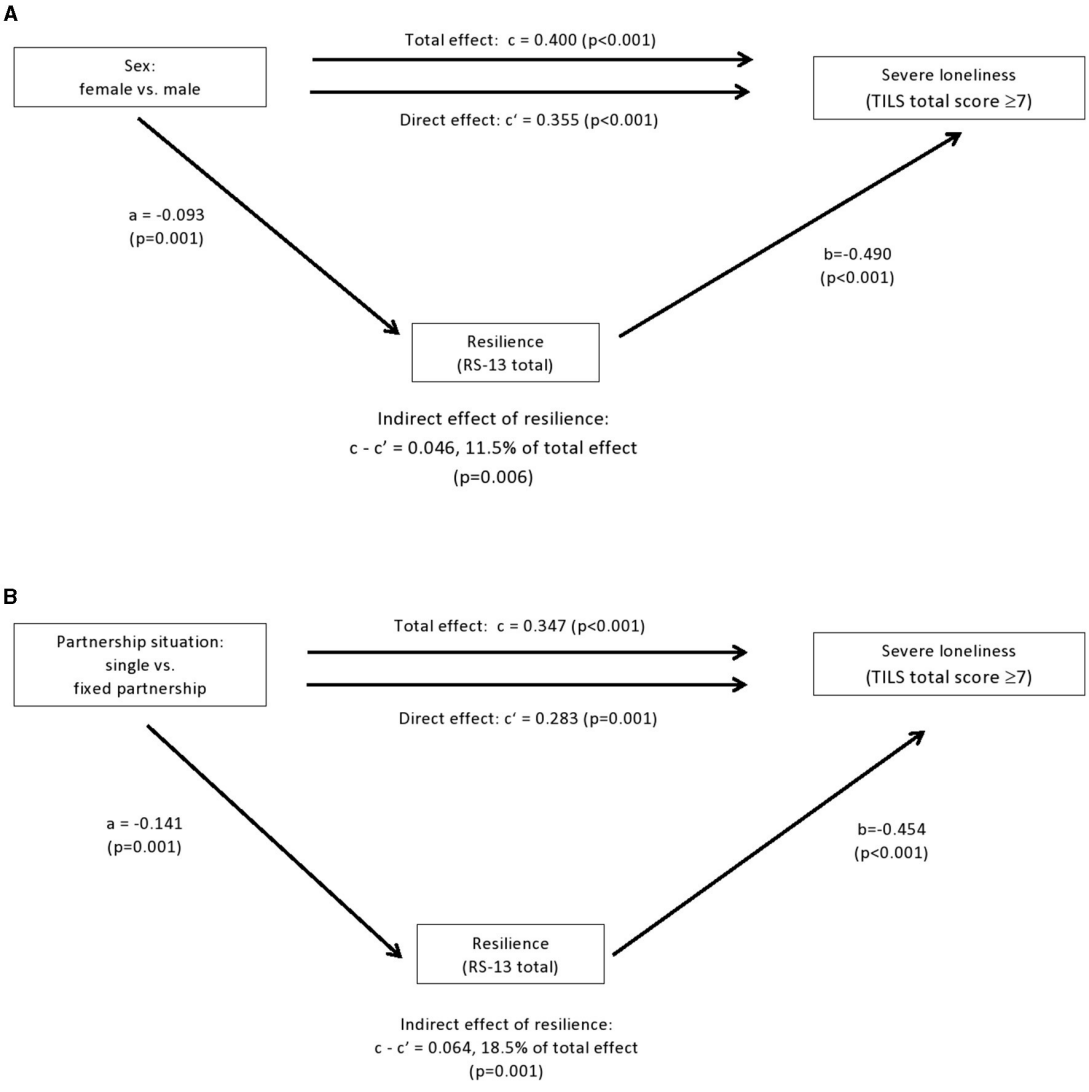
Findings of mediation analyses 2, dependent variable severe loneliness. **(A)** Effect of resilience on the relationship between sex and severe loneliness. Numbers shown are standardized regression coefficients. Solid lines indicate statistically significant effects. Extraversion (BFI total score) did not show a significant mediation effect. **(B)** Effect of resilience on the relationship between partnership situation and severe loneliness. Numbers shown are standardized regression coefficients. Solid lines indicate statistically significant effects. Extraversion (BFI total score) did not show a significant mediation effect. TILS, Three Item Loneliness Scale; RS-13, Resilience Scale.

The findings of the mediation analyses are displayed in Figures 1, 2 and in Tables A1, A2 (Supplementary Material). We first investigated to what extent the sex differences in psychological distress (higher prevalence in women, Table 2) were mediated by resilience and/or extraversion. As shown in Figure 1A, resilience emerged as a significant mediator of the sex differences (c-c' = 0.090, *p* = 0.003), whereas extraversion did not. A considerable proportion of the sex differences (35.3%) was attributable to resilience. The direct effect of sex on psychological distress lost its significance (*p* = 0.127), which may partly be a power problem, as the effect size was still rather large (c' = 0.162, 64.3% of total effect). Differences in psychological distress between study participants in a permanent partnership and singles were investigated in the same way. Both resilience and extraversion significantly mediated the effect of partnership, accounting for a proportion of 41.4% of the total effect attributable to the two mediators. The direct effect of the partnership situation on psychological distress remained significant. Regarding age, mediation analysis revealed no significant effect of either resilience or extraversion. Details can be found in Table A1.

Findings of the mediation of the effects of sex and partnership on severe loneliness are displayed in Figure 2 and Table A2. Resilience significantly mediated the relationship between sex and severe loneliness, accounting for 11.5% of the total sex difference, i.e., a comparatively small proportion. Extraversion did not show a significant mediation effect. The direct effect of sex on severe loneliness stayed significant after adjustment for resilience. Similarly, the effect of partnership on severe loneliness was significantly mediated by resilience (18.5% of the total difference), but not by extraversion. The direct effect of the partnership situation on loneliness remained significant. Regarding age, the mediation effect of neither resilience nor extraversion on the relationship between age group and severe loneliness attained significance (details in Table A2).

## Discussion

Focussing on potential sex differences and study participants' partnership situation, the main objective of this study was to investigate the mediating role of resilience and extraversion on psychological distress and loneliness during the COVID-19 pandemic among the general population of Tyrol, Austria. Overall, psychological distress and severe loneliness were more often detected in women and singles. In addition, they were less resilient, while men and singles presented with a lower degree of extraversion. However, effect sizes were small.

Our finding of a higher risk of suffering from negative psychological consequences in females in the context of the COVID-19 pandemic is in line with previous systematic reviews (41, 42). Earlier studies have shown that the transition to working from home in combination with household chores (43) and the increased involvement in home-schooling (44) due to the closure of daycare centers and schools resulted in additional burdens on women. Whereas, help from the family may have been a common factor prior to the COVID-19 pandemic (45), for instance through grandparents looking after a child in the afternoon, this support may no longer have been available since people aged 60 and older belong to the high-risk group for the COVID-19 infection (46). Thus, many women may have had limited access to supportive networks, which has been related to high psychological distress even before the onset of the pandemic (47). One of our pre-pandemic studies in healthy emerging adults, for example, revealed a much stronger interrelationship between the perception of social support and stress in women than in men (48). Moreover, in Austria, a higher proportion of women than men are employed in the health and social sectors, in education and training, and in the hospitality and commerce sectors (49), i.e., in fields of activity that were severely affected by the COVID-19 pandemic. In addition, a total of 80% of all part-time jobs in Austria are occupied by women (49), and 216,584 women worked in marginal employment positions in 2019 (50). Accordingly, they were not eligible for government subsidies such as the short-time allowance. This, in turn, can be expected to cause existential worries and to further increase psychological distress (51). Consequently, as a result of the pandemic, more women than men may be in need of increased social and family support in order to reduce COVID-19-related psychological distress and a subsequent risk of mental health symptomatology.

At the time of the survey, women were more prone to being affected by severe loneliness than men. This corroborates the findings of other research groups from all over the world (52–54), however, results gathered prior to the COVID-19 pandemic show that women generally report loneliness more frequently than men (55–57). During the pandemic and the thereof resulting home-office and home-schooling conditions especially young and employed women may have been confronted with the burden of an increase in household tasks (43) and may have had less time to rest as well as less time for self-care and for personal contacts, including contact by way of telephone or the internet. In older age groups, women have generally been suggested to be more likely to perceive loneliness because of lower male life expectancy (58) and the resulting premature loss of the partner, again resulting in widowhood (56), poor health (59), and financial difficulties (59). Social distancing in the context of the pandemic may further have increased the feeling of loneliness. However, whether men really feel less lonely than women is debatable. Borys and Perlmann (60), for example, have shown that men are less willing to admit loneliness than women. This may be a result of education, social demands, or stigma. Of note, studies from the United States (61, 62) and Brazil (63) have shown that loneliness did not increase during the initial phase of COVID-19, making it necessary to further evaluate the levels of loneliness and its potential consequences on mental health in the future.

As expected and in line with previous investigations, higher degrees of resilience and extraversion were associated with less psychological distress (17) and loneliness (18) among our sample. In addition, women and singles were less resilient compared to men and those living in a permanent partnership, which corroborates the above mentioned findings of our previous investigation (48) and those of other research groups (64, 65). Of note, resilience significantly mediated the relationship between both study participants' sex and partnership situation and psychological distress, accounting for one third of the total effect. To a lesser extent, resilience also mediated the relationship between sex/partnership situation and severe loneliness (11.5 and 18.5% of the total effect, respectively). Obviously, there are a number of other factors that have not been considered in our study and that have previously been shown to be relevant in terms of reduced psychological distress and loneliness, e.g., the availability of sources of social support in (66) and outside the family (48, 67, 68), perceived levels of family cohesion (67), social networks (66), active coping (68), optimism (68), positive reframing (68), purpose in life (68), job (dis)satisfaction (66), and the personal financial situation (69). Clearly, these protective factors can be expected to also apply during a pandemic.

A recent study from Nigeria revealed that the marital status affected overall mental health during the COVID-19 lockdown (70). Generally, the formation of intimate relationships can be considered a crucial developmental achievement in young adults (57), whereas a partner represents a person of trust to all age groups (59). Thus, not living in a permanent partnership has been identified as a risk factor for psychological distress (71), leading more single people to experience symptoms of depression during the COVID-19 period than those who are married or living together (72, 73). Taking into account this close relation of psychological distress to symptoms of depression (71, 74), one can hypothesize that the higher proportion of singles reporting psychological distress and severe loneliness among our sample may be an indirect indicator of a higher prevalence of depressive symptoms in this group at the time of the survey, however, this issue cannot be addressed by our data.

The reasons for feeling lonely and experiencing psychological distress go hand in hand with each other. Loneliness, both in intensity and duration, is correlated with psychological and somatic stress symptoms (75). Further evidence suggests that an increased time of loneliness is associated with a decrease in overall life satisfaction (76). Lower levels of global satisfaction, in turn, predict higher levels of perceived stress (77). One can assume that in the context of the COVID-19-related confinements, singles living alone spent a major portion of their time on their own. The avoidance of any social contact at work or in private life can cause or reinforce loneliness (57) and psychological distress (7). Unwanted withdrawal from society may have brought the desire of having a partner to the forefront of the discussion, which could have increased the psychological strain and further exacerbated issues related to loneliness.

Extraversion has previously been associated with sociability (78) and has shown the highest correlation with measures of well-being among all the big-five personality traits (79). Margolis and Lyubomirsky, for example, have demonstrated that introverts who behave extrovertly for 1 week show an increase in well-being (80), which, in turn, is negatively associated with psychological distress (81). Accordingly, our finding of a mediating effect of extraversion on the relationship between study participants' partnership situation and psychological distress is not surprising. Notably, a higher degree of extraversion has been related to an increased ability of adaptation to the COVID-19 lockdown in Spain (30) and has also been found to be a predictor of resilience (82). This resembles the positive association between extraversion and resilience found in our sample.

Extraversion has also been related to positive reinterpretation and growth as well as problem-focused coping (25). For example, highly extraverted people have been shown to find creative solutions to communicate with others (e.g., via video chat) (83). We did not investigate this issue in detail, however, our finding of less psychological distress and severe loneliness in extraverted study participants may be seen in this context.

Interestingly, study participants under the age of 30 experienced severe loneliness more frequently than older people, whereas psychological distress, resilience, and extraversion were comparable between age groups. This phenomenon of young people becoming increasingly lonely has been observed before (55) and during the COVID-19 pandemic (11, 52, 84) and is a matter of public concern. In 2020, more than 50% of young people between 20 and 24 years of age lived with their parents as did more than 20% in the age group of 25–29 years (85). Because of campus closures, many students had to move back home and to be with their parents (86). This can become a major task and burden to these individuals for multiple reasons, e.g., not meeting social expectations or parents' wishes, not being able to avoid each other, and involuntarily spending time together. Delays in academic activities, job insecurities and the resulting financial problems, lack of social contacts with peers, and disruptions in everyday life structure could make this age group especially vulnerable. Accordingly, prevention and interventions addressing these public health problems are urgently needed. Our findings suggest that resilience-fostering measures could help to decrease psychological distress and loneliness. Thus, targeted interventions such as resilience training focussing on mindfulness and cognitive behavioral skills (87) as well as physical exercise (88) with a focus on outdoor activity could be recommended. Other measures such as reactivating the social network, e.g., through social media (89), or more frequent phone calls to family members and other related people could increase positive feelings and decrease the level of worry, possibly leading to higher resilience and subsequently to lower psychological distress and loneliness.

The contradictory aspect of the current situation is that all procedures to alleviate psychological distress and loneliness that have been evaluated so far are based on social interactions and their frequency (57, 90). It is questionable whether this recommendation can be followed during a pandemic. In light of this, there is an urge to develop internet-based programs focussing on a reduction of psychological distress and loneliness, e.g., internet-based behavioral therapy concepts with the aim of increasing well-being. Importantly, our findings underscore the relevance of considering sex- and age-specific aspects in this regard. Moreover, various large-scale interventions at the societal level, such as cultural activities, (sports) club life, and civic participation should be promoted to fight loneliness, psychological burdens, and to strengthen the society.

Findings from this online survey need to be interpreted with caution due to several limitations. Because of the study design, only self-reported questionnaires could be used, which may be subject to desirability bias. We attempted to reach a heterogeneous group of the adult Tyrolean general population by using different information channels. We are aware that not all population groups had equal access to the internet and thus to the online survey. For example, older subjects could be reached less well and our study design did not include children and adolescents. A further part of the non-respondent population may not have participated in the context of softening of restrictions at the time of study conduction and clearly, our convenience sample is not representative for whole Austria. Moreover, the generalizability of our findings is limited because of the variability of stringency of the COVID-19-related confinements across place and time. A further limitation is that our sample was unbalanced in regards of gender and age group membership. Besides, we investigated psychological distress, loneliness, resilience, and extraversion amidst the pandemic, while pre-pandemic data are lacking. However, this is the first study investigating the mediating role of resilience and extraversion on psychological distress and on loneliness among the general population of Tyrol during the COVID-19 pandemic and the large sample size is a clear strength.

## Data Availability Statement

The datasets presented in this article are not readily available because of their proprietary nature or ethical concerns. Requests to access the datasets should be directed to Anna Chernova, anna.chernova@i-med.ac.at.

## Ethics Statement

The studies involving human participants were reviewed and approved by Ethics Committee Medical University Innsbruck. Written informed consent for participation was not required for this study in accordance with the national legislation and the institutional requirements.

## Author Contributions

AH, BF-A, SP, BH, and BP designed the study and wrote the protocol. Recruitment was performed by AC and FT. GK undertook statistical analysis. AC wrote the first draft of the manuscript. All authors have contributed to approved the final manuscript.

## Funding

This work was supported by a grant (no. F.21427) from the federal state of Tyrol.

## Conflict of Interest

BH owns part of the IPRs of the CHES software tool. The remaining authors declare that the research was conducted in the absence of any commercial or financial relationships that could be construed as a potential conflict of interest.

## Publisher's Note

All claims expressed in this article are solely those of the authors and do not necessarily represent those of their affiliated organizations, or those of the publisher, the editors and the reviewers. Any product that may be evaluated in this article, or claim that may be made by its manufacturer, is not guaranteed or endorsed by the publisher.
