# Supplementary material for: The Mediating Role of Resilience and Extraversion on Psychological Distress and Loneliness Among the General Population of Tyrol, Austria Between the First and the Second Wave of the COVID-19 Pandemic
Source: Front Psychiatry. 2021 Oct 27;12:766261. doi: 10.3389/fpsyt.2021.766261 (PMC8578839; doi:10.3389/fpsyt.2021.766261)
Supplement: Supplementary file 1 [file Data_Sheet_1.docx]

**Tables for Appendix**

**Table A1: Mediation of the effects of sex, partnership and age group on psychological distress (GSI) by resilience and extraversion**

| Independent variable, X | Effect sizes | | | | | |
| --- | --- | --- | --- | --- | --- | --- |
|  | Total effect (c) | Direct effect (c’) | Parameter a  (X → mediator) | Parameter b (mediator → GSI) | Mediation effect (a⋅b) with 95% CI | % of total effect mediated |
| Sex (female vs. male) | 0.252**  p = 0.003 | 0.162  p=0.127 |  |  |  |  |
| Mediator resilience |  |  | - 0.094**  p = 0.003 | -0.952**  p < 0.001 | 0.090**  p = 0.003 | 35.7% |
| *Mediator extraversion^a^* |  |  | *0.043*  *p = 0.186 n.s.* | *-0.322***  *p = 0.003* | *-0.014*  *p = 0.254 n.s.* | *-* |
| Partnership situation (single vs.   partnership) | 0.331**  p < 0.001 | 0.194*  p=0.034 |  |  |  |  |
| Mediator resilience |  |  | - 0.142**  p < 0.001 | -0.805**  p < 0.001 | 0.115**  p = 0.001 | 34.7% |
| Mediator extraversion |  |  | - 0.074*  p = 0.019 | -0.300**  p = 0.005 | 0.022**  p = 0.048 | 6.7% |
| Age group (18-29 vs. 30-59 vs. 60-96 years)^b^ | -0.065  p=0.549 n.s. | -0.120  p=0.229 n.s. |  |  |  |  |
| *Mediator resilience ^b^* |  |  | *-0.048*  *p = 0.135 n.s.* | *-0.831*  *p<0.001*** | *0.040*  *p = 0.157 n.s.* | *-* |
| *Mediator extraversion* *^b^* |  |  | *-0.049*  *p = 0.130 n.s.* | *-0.320**  *p = 0.033* | *0.015*  *p = 0.226 n.s.* | *-* |

^a^ Extraversion was not significant and hence excluded from the final model

^b^ Neither the total effect nor the direct effect of age group on psychological distress nor the mediation effects of resilience or extraversion attained significance.

**Table A2: Mediation of the effects of sex, partnership and age group on severe loneliness (TILS score ≥ 7) by resilience and extraversion**

| Independent variable, X | Effect sizes | | | | | |
| --- | --- | --- | --- | --- | --- | --- |
|  | Total effect (c) | Direct effect (c’) | Parameter a  (X → mediator) | Parameter b (mediator → GSI) | Mediation effect (a⋅b) with 95% CI | % of total effect mediated |
| Sex (female vs. male) | 0.400**  p < 0.001 | 0.355**  p < 0.001 |  |  |  |  |
| Mediator resilience |  |  | - 0.093**  p = 0.003 | -0.490**  p < 0.001 | 0.046**  p = 0.006 | 11.5% |
| *Mediator extraversion ^a^* |  |  | *0.043*  *p = 0.186 n.s.* | *-0.322***  *p = 0.003* | *-0.014*  *p = 0.254 n.s.* | *-* |
| Partnership situation (single vs.   partnership) | 0.347**  p < 0.001 | 0.283**  p=0.001 |  |  |  |  |
| Mediator resilience |  |  | - 0.141**  p = 0.001 | -0.454**  p < 0.001 | 0.064**  p = 0.001 | 18.5% |
| *Mediator extraversion ^a^* |  |  | *- 0.074**  *p = 0.019* | *0. 150*  *p = 0.894* | *-0.001*  *p = 0.883 n.s.* | *-* |
| Age group (18-29 vs. 30-59 vs. 60-96 years) | -0.184*  p=0.027 | -0.207*  p=0.010 |  |  |  |  |
| *Mediator resilience^b^* |  |  | *-0.047*  *p = 0.143* | *-0.495***  *p < 0.001* | *0.023*  *p = 0.174 n.s.* |  |
| *Mediator extraversion^b^* |  |  | *-0.050*  *p = 0.118* | *-0.009*  *p = 0.922* | *0.001*  *p = 0.944 n.s.* |  |

^a^ Extraversion was not significant and therefore excluded from the final model

^b^ Neither resilience nor extraversion significantly mediated the relationship between age group and severe loneliness.
